# Sex-Specific Associations of Glycemic Status and Smoking with Bladder Cancer Risk: A Nationwide Cohort Study

**DOI:** 10.3390/cancers17132262

**Published:** 2025-07-07

**Authors:** Joo-Hyun Park, Jung Yong Hong, Kyungdo Han, Jay J. Shen

**Affiliations:** 1 Department of Family Medicine, Korea University Ansan Hospital, Korea University College of Medicine, Ansan 15355, Republic of Korea; joohyun_park@korea.ac.kr; 2 Department of Healthcare Administration and Policy, School of Public Health, University of Nevada Las Vegas, Las Vegas, NV 89154, USA; 3 Division of Hematology-Oncology, Department of Medicine, Samsung Medical Center, Sungkyunkwan University School of Medicine, Seoul 06351, Republic of Korea; 4 Department of Clinical Research Design and Evaluation, Samsung Advanced Institute for Health Science and Technology (SAIHST), Sungkyunkwan University, Seoul 06351, Republic of Korea; 5 Department of Statistics and Actuarial Science, Soongsil University, Seoul 06978, Republic of Korea; hkd917@naver.com

**Keywords:** bladder cancer, diabetes, prediabetes, smoking, sex differences

## Abstract

**Background**: Sex differences in the effects of hyperglycemia and smoking on bladder cancer risk remain poorly understood, despite their known roles as modifiable risk factors. We investigated the sex-specific associations of prediabetes, diabetes, and smoking with bladder cancer risk. **Methods**: We analyzed data from 9,492,331 cancer-free adults (54.8% men) who underwent the 2009 Korean national health screening. Adjusted hazard ratios (aHRs) and 95% confidence intervals (CIs) for bladder cancer incidence were calculated using Cox proportional hazards models. **Results**: Over a median follow-up of 8.3 years, 12,095 men and 2467 women were diagnosed with bladder cancer. The male-to-female incidence ratio was 4.1:1 among never-smokers with normoglycemia and 2.7:1 among ever-smokers with diabetes. In women, both prediabetes and diabetes were associated with elevated bladder cancer risk (aHRs, 95% CIs: 1.12, 1.02–1.24; and 1.27, 1.13–1.43). In men, only diabetes showed an increased risk (aHR: 1.22, 1.12–1.32). Combined diabetes and smoking increased the risk synergistically in women (aHR: 2.75, 1.95–3.87; synergy index = 2.38, *p* < 0.01), while the effect was additive in men (aHR: 1.82, 1.70–1.95). **Conclusions**: The typical male predominance in bladder cancer incidence appeared attenuated in the presence of hyperglycemia and smoking, suggesting that these risk factors may have a relatively greater impact on bladder cancer risk among women. These findings underscore the importance of targeted bladder cancer prevention strategies, with particular attention to women with hyperglycemia who smoke, given their disproportionately elevated risk.

## Simple Summary

Bladder cancer occurs more frequently in men, yet the sex-specific effects of hyperglycemia and smoking on bladder cancer risk remain poorly understood, despite their established roles as modifiable risk factors. In this large, nationwide cohort study of over nine million Korean adults, we investigated the sex-specific associations of prediabetes, diabetes, and smoking with the risk of developing bladder cancer. The results showed that women exhibited a linear increase in risk with worsening glycemic status, whereas men demonstrated an apparent threshold pattern. Moreover, the combination of hyperglycemia and smoking synergistically elevated bladder cancer risk in women, while the effect was additive in men. Notably, the typical male predominance in bladder cancer incidence was attenuated in the presence of these risk factors. These findings suggest that women may be more vulnerable to the carcinogenic effects of hyperglycemia on the bladder, particularly when combined with smoking. Although the overall incidence is lower in women, those with both hyperglycemia and smoking exposure face a disproportionately high risk, underscoring the need for targeted education and prevention efforts in this vulnerable group.

## 1. Introduction

Bladder cancer ranks as the 10th most commonly diagnosed malignancy worldwide, with an estimated 573,000 new cases reported in 2020 [1,2,3,4]. In South Korea, bladder cancer accounted for 1.9% of all incident cancers in 2022, ranking 10th among men and 14th among women, with a 38% increase in incidence between 2010 and 2019 [5,6]. The incidence rises sharply with age, with most cases diagnosed in individuals aged 70 years and older [7]. Men exhibit a significantly higher incidence than women, with a global male-to-female ratio of approximately 4:1 [8]. Given the high immunogenicity of bladder cancer, this disparity may be partly attributed to sex-based immunological differences [9,10]. Women are known to exhibit more robust innate and adaptive immune responses, which may enhance immune surveillance and reduce susceptibility to tumorigenesis [11,12,13]. These differences highlight the potential for sex-specific variations in the influence of risk factors related to immune dysfunction on bladder cancer development.

Smoking is a well-established risk factor for bladder cancer in both sexes, contributing to immune suppression and impaired inflammatory regulation [14,15,16]. Likewise, hyperglycemia disrupts immune homeostasis and has been associated with increased bladder cancer risk [17,18,19,20,21]. However, the sex-specific dose–response relationship between glycemic status and bladder cancer risk remains unclear. Previous studies have reported inconsistent findings, with positive associations observed in men [18,19], in women [21,22], or without sex-stratified analyses [20]. These studies have often been limited by small sample sizes, reliance on self-reported diabetes diagnoses, and inadequate adjustment for key confounders, including smoking, obesity, and chronic kidney disease (CKD) [18,19,20,21]. Moreover, the potential interaction between hyperglycemia and smoking in relation to bladder cancer risk by sex has not been thoroughly examined.

In the Korean adult population, the prevalence of diabetes mellitus among individuals aged ≥ 30 years was 15.5% in 2021–2022, with a higher prevalence in men (18.1%) than in women (13.0%) [23]. In 2022, the prevalence of tobacco use was 36.6% among men and 7.2% among women, indicating a substantial sex-based disparity [24].

To address these gaps, we conducted a nationwide cohort study to evaluate sex-specific differences in the independent and combined associations of glycemic status and smoking with bladder cancer risk. Using data from over 9 million cancer-free adults who underwent standardized health screening, we integrated laboratory test results and self-reported health-related behaviors, with follow-up extending up to 10 years.

## 2. Methods

### 2.1. Data Source and Study Population

The Korean National Health Insurance Service (KNHIS) operates as a universal, single-payer system covering approximately 97% of the national population. It offers a standardized biennial health screening program to all insured individuals aged ≥ 20 years and to all employees regardless of age, with an overall participation rate of approximately 76% among eligible individuals [25]. The dataset used in this study included results from health screenings—comprising laboratory tests, anthropometric measurements, and self-reported health behavior questionnaires—with insurance claims records encompassing diagnoses, treatments, and prescriptions coded according to the International Classification of Diseases, 10th Revision, Clinical Modification (ICD-10-CM).

**Figure 1:**
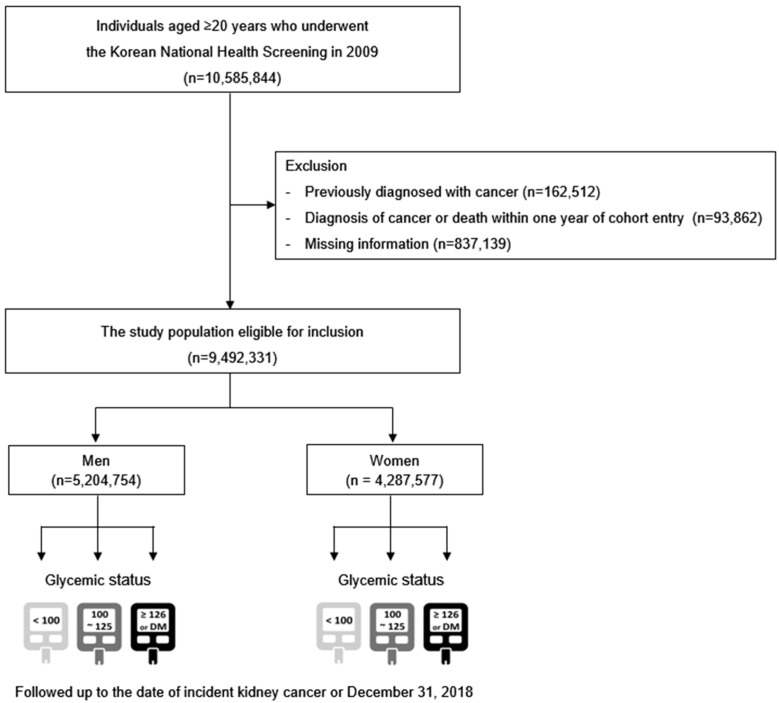
Study population selection flowchart.

Figure 1 presents a flowchart of the cohort selection process. Of 10,585,844 adults aged ≥ 20 years who underwent health screening between 1 January and 31 December 2009, we excluded individuals with a history of cancer prior to cohort entry (*n* = 162,512), those who developed cancer or died within the first year of follow-up to reduce reverse causation and detection bias (*n* = 93,862), and those with missing data (*n* = 837,139). The final cohort included 9,492,331 individuals who were followed from the date of screening until the occurrence of bladder cancer, death, or 31 December 2018, whichever came first. The median age of participants at baseline was 47.4 years (interquartile range: 38.0–56.9).

This study received ethical approval from the Institutional Review Board of Samsung Medical Center, Seoul, South Korea (approval number: SMC2022-10-030), as well as from the Review Committee of the KNHIS Big Data Steering Department (approval number: NHIS-2024-1-326). Owing to the retrospective nature of the study and the use of anonymized, de-identified data, the requirement for informed consent was waived. All study procedures complied with the ethical standards outlined in the Declaration of Helsinki.

### 2.2. Definitions of Variables

#### 2.2.1. Glycemic Status

Fasting plasma glucose (FPG) levels were obtained through the routine procedures of the national health screening program. Certified healthcare professionals at KNHIS-affiliated institutions collected blood samples following an overnight fast. Glycemic status was categorized into three groups: normoglycemia (fasting glucose < 100 mg/dL), prediabetes (100–125 mg/dL), and diabetes (fasting glucose ≥ 126 mg/dL or ≥1 insurance claim per year for antidiabetic medications, either oral or injectable, under ICD-10-CM codes E11–E14) [26,27,28]. The definition of prediabetes was based on impaired fasting glucose criteria established by the American Diabetes Association, which are widely used in large-scale epidemiologic research [26,27,28].

#### 2.2.2. Incident Bladder Cancer

The primary outcome was the incidence of newly diagnosed bladder cancer between January 2009 and December 2018. Cases were identified through hospitalization records using ICD-10-CM code C67 and the special reimbursement code V193, which is issued by the KNHIS upon physician-certified cancer diagnosis. Since 2006, this certification has been required to grant patients eligibility for reduced co-payments on cancer-related medical services, thereby ensuring the diagnostic accuracy of cancer cases. The validity of this claims-based definition has been supported by previous studies [27,29].

#### 2.2.3. Clinical Variables

Health-related behaviors were assessed using a standardized, self-administered questionnaire. Smoking status was classified as never-smoker (<100 lifetime cigarettes) or ever-smoker. Ever-smokers were further stratified by cumulative tobacco exposure using pack-years, calculated by multiplying the number of cigarette packs smoked per day by the number of smoking years. Alcohol intake was classified into three categories: none, light-to-moderate (<30 g/day), and heavy (≥30 g/day) [30]. Participants were considered physically active if they performed vigorous exercise for 20 min or more on three or more days per week, or moderate exercise for 30 min or more on five or more days weekly [31].

Measurements of height, weight, and waist circumference were obtained as part of the anthropometric assessment. Body mass index (BMI) was calculated as weight (kg) divided by height squared (m^2^). Blood pressure was assessed after participants remained seated and rested for five minutes. Laboratory tests included serum levels of total cholesterol, low-density lipoprotein (LDL) cholesterol, high-density lipoprotein (HDL) cholesterol, and creatinine. The estimated glomerular filtration rate (eGFR) was calculated using the Modification of Diet in Renal Disease (MDRD) equation, and chronic kidney disease (CKD) was defined as an eGFR < 60 mL/min/1.73 m^2^ [32,33].

Dyslipidemia was defined as either a total cholesterol concentration of ≥240 mg/dL or the prescription of lipid-lowering agents corresponding to ICD-10-CM code E78. Hypertension was classified based on a systolic blood pressure ≥ 140 mmHg, a diastolic blood pressure ≥ 90 mmHg, or use of antihypertensive drugs recorded under ICD-10-CM codes I10–I13 and I15. Individuals were classified as having low income if they belonged to the lowest quartile of health insurance contributions or were beneficiaries of government-funded medical assistance programs. A diagnosis of urinary tract infection was established according to ICD-10-CM code N39.0.

### 2.3. Statistical Analysis

Comparisons of baseline characteristics were conducted with independent t-tests for continuous measures and chi-square tests for categorical data. Age-standardized bladder cancer incidence rates (IRs) were expressed as cases per 100,000 person-years. Analyses stratified by sex were conducted to examine the relationship between glycemic status and bladder cancer risk across different smoking categories.

Hazard ratios (HRs) and 95% confidence intervals (CIs) for incident bladder cancer were estimated using multivariable-adjusted Cox proportional hazards regression models. Three models were developed sequentially: Model 1 adjusted for age; Model 2 additionally adjusted for alcohol consumption and physical activity; and Model 3 further adjusted for hypertension, CKD, dyslipidemia, urinary tract infection, and BMI.

To assess interaction effects between glycemic status and smoking, we calculated the synergy index (S), which quantifies departure from additivity. The index was defined as: S = [HR_A+B+_ − 1]/(HR_A+B−_ − 1) + (HR_A−B+_ − 1), where HR_A+B+_ equals the HR of the combined effect of the two risk factors, while HR_A+B−_ and HR_A−B+_ equals the HR of each risk factor alone [34]. An S value of 1 indicates no interaction (additive effect), values > 1 indicate a positive (synergistic) interaction, and values < 1 indicate a negative (sub-additive) interaction. We also calculated the relative excess risk due to interaction (RERI) to further evaluate additive interaction. RERI was computed as: RERI = HR_AB_ − HR_A_ − HR_B_ + 1, where RERI = 0 denotes no interaction (additive effect), RERI > 0 indicates a positive (synergistic) interaction, and RERI < 0 reflects a negative (sub-additive) interaction [34,35]. All statistical tests were two-sided, with a significance threshold set at *p* < 0.05. Analyses were conducted using SAS software (version 9.3; SAS Institute, Cary, NC, USA).

## 3. Results

### 3.1. Participants’ Baseline Characteristics

Among 9,492,331 participants (54.8% men), 14,562 cases of newly diagnosed bladder cancer were identified—12,095 in men and 2467 in women—over a median follow-up of 8.3 years (78.1 million person-years). The mean age at baseline was 45.5 years (SD 13.3) for men and 48.9 years (SD 14.3) for women. Table 1 summarizes baseline characteristics stratified by sex and bladder cancer status. Across both sexes, those who developed bladder cancer were significantly older and had higher FPG levels than those without cancer (all *p* < 0.01). Moreover, the prevalence of diabetes, dyslipidemia, hypertension, and CKD was significantly higher among participants with bladder cancer (all *p* < 0.01).

Baseline characteristics according to glycemic status by sex are presented in Supplemental Table S1. The prevalence of prediabetes and diabetes was 25.7% and 9.7% in men, and 19.2% and 7.5% in women, respectively. Participants with diabetes were older and more frequently reported abstaining from alcohol consumption and engaging in regular physical activity (all *p* < 0.01).

### 3.2. Association of Glycemic and Smoking Status with Bladder Cancer Risk in Men and Women

Supplementary Table S2 presents the association between glycemic status, smoking status, and bladder cancer risk in the overall study population, without stratification by sex. In this multivariable-adjusted analysis, diabetes (HR 1.20, 95% CI 1.12–1.29), former smoking (HR 1.28, 95% CI 1.20–1.36), and current smoking (HR 1.71, 95% CI 1.61–1.81) were independently associated with an increased risk of bladder cancer, whereas prediabetes was not (HR 1.03, 95% CI 0.97–1.10).

Table 2 displays the association between glycemic status, smoking status, and bladder cancer risk stratified by sex. Age-standardized IRs of bladder cancer according to glycemic and smoking status are also presented for men and women. Across all categories, men exhibited higher bladder cancer IRs than women. However, sex differences in IRs narrowed with increasing exposure to hyperglycemia and smoking. Among never-smokers with normoglycemia, the male-to-female IR ratio was 4.1 (33.7 vs. 8.2 per 100,000 person-years in men and women, respectively), which decreased to 2.7 among ever-smokers with diabetes (63.5 vs. 23.1 per 100,000 person-years, respectively).

**Figure 2:**
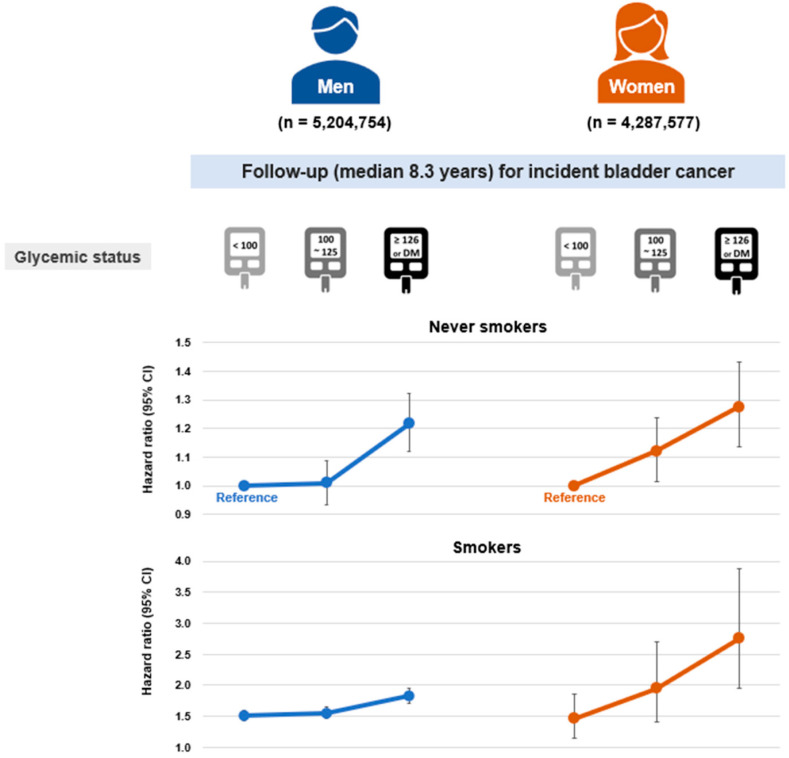
Dose–response association between glycemic status and bladder cancer risk according to sex in never-smokers and ever-smokers. Hazard ratios and 95% confidence intervals were obtained for the models adjusted for age, sex, body mass index, alcohol consumption, physical activity, hypertension, dyslipidemia, chronic kidney disease, and urinary tract infection.

Among never-smokers, diabetes was linked to a significantly elevated risk of bladder cancer in men (Model 3; HR = 1.22, 95% CI: 1.12–1.32), whereas prediabetes did not show a significant association (HR: 1.01, 95% CI: 0.94–1.09; Table 2). In women, however, both prediabetes and diabetes were significantly associated with increased bladder cancer risk, demonstrating a linear dose–response gradient (HR: 1.12, 95% CI: 1.02–1.24; and HR: 1.27, 95% CI: 1.14–1.43, respectively; *p* for trend < 0.01). An apparent threshold effect was observed in men, while a more continuous dose–response trend was noted in women, although statistical significance was not consistent across all exposure categories (Figure 2).

When combined with smoking, diabetes was associated with a synergistically increased risk of bladder cancer in women (HR: 2.75, 95% CI: 1.95–3.87; synergy index = 2.38, *p* < 0.01), whereas in men, the combined effect was additive without evidence of synergy (HR: 1.82, 95% CI: 1.70–1.95). Among women, the combination of smoking and prediabetes also showed a synergistic effect (HR: 1.95, 95% CI: 1.40–2.70; synergy index = 1.62, *p* < 0.01).

### 3.3. Independent and Combined Associations of Glycemic Status and Smoking with Bladder Cancer Risk

**Figure 3:**
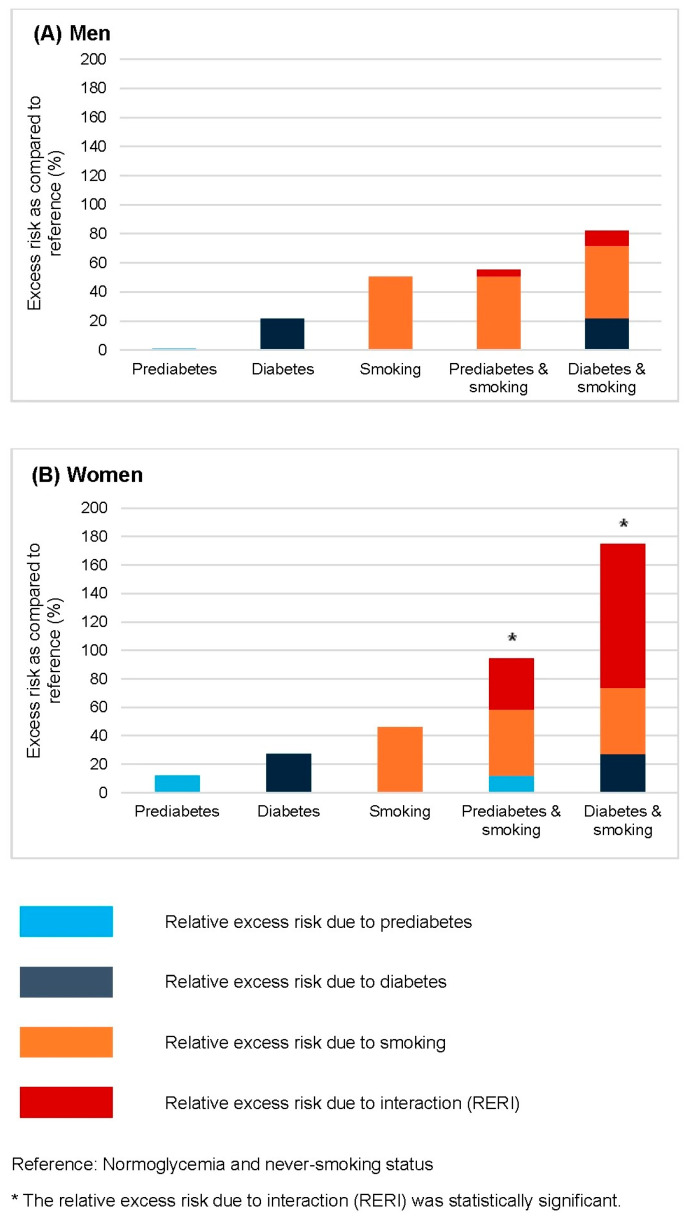
The relative excess risk for bladder cancer conferred by smoking combined with prediabetes and diabetes in men (**A**) and women (**B**), based on Model 3. The model was adjusted for age, body mass index, alcohol consumption, physical activity, hypertension, dyslipidemia, urinary tract infection, and chronic kidney disease. The reference group comprised individuals with normoglycemia and no history of smoking, within each sex stratum. Bars represent the relative excess risk from prediabetes, diabetes, smoking, and their interactions. * Significant positive interactions are defined as relative excess risks due to interaction greater than 0 (*p* < 0.01).

Figure 3 illustrates the relative excess risk of bladder cancer associated with the combination of prediabetes or diabetes and smoking, stratified by sex (Figure 3A: men; Figure 3B: women). Additive interactions were assessed using relative excess risk due to interaction (RERI), with normoglycemic never-smokers serving as the reference group. In men, no significant interaction was observed between glycemic status and smoking in relation to bladder cancer risk. In contrast, women showed significant additive interactions: the combination of prediabetes and smoking yielded a RERI of 0.36, while the combination of diabetes and smoking resulted in a RERI of 1.01 (both *p* < 0.001), indicating that the joint effects exceeded the sum of individual risks.

### 3.4. Sex-Specific Associations of Glycemic Status and Smoking Intensity with Bladder Cancer Risk

Supplementary Figure S1 presents age-standardized incidence rates of bladder cancer stratified by glycemic status and smoking pack-years. The male-to-female incidence ratio decreased with worsening glycemic status and increasing smoking exposure: from 4.1:1 in never-smokers with normoglycemia to 1.5:1 in those with diabetes and ≥20 pack-years of smoking. As shown in Supplementary Table S3, when both diabetes and ≥20 pack-years of smoking were present, the incidence rate was 68.9 per 100,000 person-years in men and 44.5 in women. This combination was associated with an additively increased bladder cancer risk in men (HR: 1.99, 95% CI: 1.85–2.15), but a synergistically elevated risk in women (HR: 5.16, 95% CI: 3.19–8.34; synergy index = 2.70, *p* < 0.01).

## 4. Discussion

In this nationwide cohort study involving over 9 million cancer-free adults, we observed a linear dose–response relationship between glycemic status and bladder cancer risk in women, whereas an apparent threshold pattern was noted in men. Moreover, the combination of hyperglycemia and smoking was associated with a synergistic increase in bladder cancer risk among women, but only an additive effect among men. Importantly, the male predominance in bladder cancer incidence was attenuated in the presence of hyperglycemia and smoking. This study provides epidemiological evidence that women may be more susceptible than men to the harmful effects of hyperglycemia on bladder cancer risk, particularly when combined with smoking.

Despite smoking being a well-established risk factor for bladder cancer in both sexes, sex differences in the impact of glycemic status remain uncertain. Previous studies have yielded inconsistent results, reporting positive associations among men [18,19], among women [21,22], or in analyses that did not stratify by sex [20]. The Netherlands Cohort Study reported an association between diabetes and invasive bladder cancer only in women, 2.19 (95% CI 1.10–4.34), but not in men, 1.42 (95% CI 0.88–2.30), although the interaction by sex did not reach statistical significance [22]. In contrast, a meta-analysis of 36 observational studies reported a modest increase in bladder cancer risk among men (RR 1.38, 95% CI 1.08–1.78) but not among women (RR 1.38, 95% CI 0.90–2.10) [18].

These inconsistencies may be attributed to differences in study design, population characteristics, and methods used to ascertain glycemic status. While numerous prior studies relied on self-reported diabetes, which is prone to misclassification bias, our study utilized objectively measured fasting plasma glucose levels. Moreover, earlier investigations often lacked adequate adjustment for key confounding variables—such as smoking, BMI, CKD, and urinary tract infections—whereas our analysis comprehensively accounted for these factors. In addition, the case–control design frequently employed in previous studies is susceptible to recall, selection, and survivorship biases. Additionally, the low prevalence of smoking among Korean women may have reduced potential confounding by smoking, thereby allowing the independent impact of hyperglycemia to be more clearly observed. To our knowledge, this is the first large-scale cohort study to evaluate the interaction between hyperglycemia and smoking on bladder cancer risk by sex. By leveraging a nationally representative cohort with objectively measured glycemic data and thorough adjustment for major confounders, our findings may provide novel insights into population-specific patterns of bladder cancer risk.

Several biological mechanisms may explain the observed associations. Hyperglycemia may contribute to bladder carcinogenesis through impaired immune function, chronic inflammation, and activation of insulin and IGF-1 signaling pathways [36,37,38]. Women, who typically have stronger baseline immune surveillance, may be more susceptible to immune disruption by hyperglycemia and smoking. These exposures may jointly impair immunological defenses, leading to a disproportionate increase in bladder cancer risk in women. Synergistic effects may also involve increased oxidative stress, DNA damage, and cytotoxicity [15,16,17,39,40].

This study has multiple strengths. First, it represents one of the largest population-based cohort studies to date, including over 9 million participants and a 10-year follow-up period, enabling robust estimation of bladder cancer risk across sex and exposure subgroups. Second, clinical outcomes were accurately captured through the KNHIS database, with high case ascertainment particularly among women and never-smokers. Third, glycemic status was determined using objective criteria, including FPG levels, diagnostic codes, and prescriptions, thereby minimizing recall bias. Fourth, we adjusted for a comprehensive set of confounders, including BMI, CKD, and urinary tract infection.

Several limitations should also be acknowledged. First, the database lacked information on the family history of bladder cancer, which may have provided additional insight into individual risk. Second, data on hemoglobin A1c levels were unavailable, limiting the ability to comprehensively define glycemic status. In addition, glycemic status was assessed only at baseline, and changes during the follow-up period were not taken into account. Third, information on occupational exposures to established bladder carcinogens—such as benzidine dyes and industrial chemicals—was not available. Fourth, pathological subtypes of bladder cancer were not distinguished; however, urothelial carcinoma constitutes more than 90% of all cases [41]. Finally, the study did not account for ethnic differences in bladder cancer risk, highlighting the need for further studies in more diverse populations.

## 5. Conclusions

This nationwide cohort study of over 9 million cancer-free adults demonstrated sex-specific associations of glycemic status and smoking with bladder cancer risk. Women exhibited a linear dose–response relationship between glycemic status and bladder cancer risk, whereas men showed an apparent threshold pattern. The combination of hyperglycemia and smoking was associated with a synergistic increase in bladder cancer risk among women and an additive effect among men. Notably, the typical male predominance in bladder cancer incidence appeared attenuated in the presence of these risk factors. These findings suggest that women may be more susceptible than men to the carcinogenic effects of hyperglycemia on the bladder, particularly when combined with smoking. Healthcare providers should recognize the substantially elevated risk of bladder cancer among women with these risk factors and provide tailored, proactive education to support prevention efforts. Given these observations, further studies with extended follow-up and repeated assessments of glycemic status may help clarify temporal relationships and strengthen causal inference. Additionally, further research is warranted to elucidate the sex-specific biological mechanisms underlying these associations.

## Figures and Tables

**Table 1:** Baseline characteristics of the study population.

	Bladder Cancer					
	Men			Women		
	No	Yes	*p* Value	No	Yes	*p* Value
	(n = 5,192,659)	(n = 12,095)		(n = 4,285,110)	(n = 2467)	
Age (years), mean ± SD	45.5 ± 13.5	62.6 ± 10.9	<0.001	48.9 ± 14.5	63.9 ± 11.2	<0.001
Anthropometrics, mean ± SD						
Body mass index (kg/m^2^)	24.2 ± 3.3	24.0 ± 2.9	<0.001	23.2 ± 3.5	24.4 ± 3.4	<0.001
Waist circumference (cm)	83.6 ± 8.2	85.2 ± 7.9	<0.001	76.3 ± 9.3	81.0 ± 9.0	<0.001
Systolic BP (mmHg)	124.7 ± 14.1	128.7 ± 15.6	<0.001	119.9 ± 15.7	127.1 ± 16.7	<0.001
Diastolic BP (mmHg)	78.1 ± 9.7	78.8 ± 10.1	<0.001	74.3 ± 10.1	77.0 ± 10.3	<0.001
Laboratory findings, mean ± SD						
Fasting glucose (mg/dL)	99.0 ± 25.7	105.2 ± 30.2	<0.001	95.2 ± 21.2	101.6 ± 26.1	<0.001
Total cholesterol (mg/dL)	194.8 ± 41.1	193.7 ± 37.3	0.005	196.4 ± 42.1	205.4 ± 38.3	<0.001
HDL-cholesterol (mg/dL)	53.8 ± 32.1	53.3 ± 35.8	0.069	60.1 ± 34.7	56.4 ± 28.5	<0.001
LDL-cholesterol (mg/dL)	111.9 ± 39.2	112.3 ± 42.8	0.292	115.6 ± 38.5	123.0 ± 35.4	<0.001
eGFR (mL/min/1.73 m^2^)	87.9 ± 49.5	82.8 ± 40.0	<0.001	87.1 ± 34.9	80.3 ± 33.5	<0.001
Smoking status, n (%)			<0.001			<0.001
Never	1,600,651 (30.8)	3680 (30.4)		4,073,380 (95.1)	2325 (94.2)	
Ever	3,592,008 (69.2)	8415 (69.6)		211,730 (4.9)	142 (5.8)	
Alcohol consumption, n (%)			<0.001			<0.001
None	1,535,578 (29.6)	5153 (42.6)		3,164,695 (73.9)	2174 (88.1)	
Light-to-moderate	2,924,163 (56.3)	5363 (44.3)		1,071,479 (25.0)	277 (11.2)	
Heavy	732,918 (14.1)	1579 (13.1)		48,936 (1.1)	16 (0.7)	
Regular exercise, n (%)	1,044,343 (20.1)	2948 (24.4)	<0.001	669,363 (15.6)	425 (17.2)	0.028
Low-income status, n (%)	768,416 (14.8)	2095 (17.3)	<0.001	898,788 (21.0)	456 (18.5)	0.002
Comorbidities, n (%)						
Diabetes	502,022 (9.7)	2432 (20.1)	<0.001	319,270 (7.5)	441 (17.9)	<0.001
Hypertension	1,380,713 (26.6)	6075 (50.2)	<0.001	1,064,170 (24.8)	1246 (50.5)	<0.001
Dyslipidemia	861,852 (16.6)	2992 (24.7)	<0.001	864,327 (20.2)	887 (36.0)	<0.001
Chronic kidney disease	307,675 (5.9)	1317 (10.9)	<0.001	338,250 (7.9)	437 (17.7)	<0.001

BP, blood pressure; eGFR, estimated glomerular filtration rate; HDL, high-density lipoprotein; LDL, low-density lipoprotein; SD, standard deviation.

**Table 2:** Association of glycemic and smoking status with bladder cancer risk in men and women.

Glycemic Status	n	Event, n	Person-Years	Age-Standardized IR *	Hazard Ratio (95% CI)		
					Model 1	Model 2	Model 3
**Men**							
Never-smokers							
Normoglycemia	1,042,497	1892	8,588,613	33.7	1 (Reference)	1 (Reference)	1 (Reference)
Prediabetes	404,475	1024	3,306,484	34.5	1.03 (0.95–1.11)	1.03 (0.95–1.11)	1.01(0.94–1.09)
Diabetes	157,359	764	1,240,844	42.7	1.28 (1.17–1.39)	1.28 (1.17–1.39)	1.22 (1.12–1.32)
Ever-smokers							
Normoglycemia	2,318,334	4364	19,088,353	50.0	1.49 (1.41–1.57)	1.50 (1.42–1.59)	1.50 (1.42–1.59)
Prediabetes	934,994	2383	7,645,080	52.5	1.56 (1.47–1.66)	1.58 (1.48–1.68)	1.55 (1.46–1.65)
Diabetes	347,095	1668	2,760,009	63.5	1.90 (1.78–2.03)	1.91 (1.79–2.04)	1.82 (1.70–1.95)
**Women**							
Never-smokers							
Normoglycemia	2,985,479	1339	24,789,957	8.2	1 (Reference)	1 (Reference)	1 (Reference)
Prediabetes	784,826	579	6,490,479	9.4	1.15 (1.04–1.27)	1.15 (1.04–1.27)	1.12 (1.02–1.24)
Diabetes	305,400	407	2,473,012	11.1	1.36 (1.22–1.53)	1.36 (1.21–1.52)	1.27 (1.14–1.43)
Ever-smokers							
Normoglycemia	159,320	71	1,313,052	11.5	1.42 (1.12–1.80)	1.46 (1.15–1.86)	1.46 (1.15–1.86)
Prediabetes	38,241	37	312,425	15.6	1.93 (1.39–2.68)	1.99 (1.44–2.77)	1.95 (1.40–2.70)
Diabetes	14,311	34	113,398	23.1	2.87 (2.04–4.04)	2.92 (2.08–4.12)	2.75 (1.95–3.87)

* Age-standardized incidence rate of bladder cancer per 100,000 person-years. Model 1: adjusted for age. Model 2: adjusted for age, alcohol consumption, and physical activity. Model 3: adjusted for age, alcohol consumption, physical activity, body mass index, hypertension, dyslipidemia, chronic kidney disease, and urinary tract infection. CI, confidence interval; IR, incidence rate.

## Data Availability

The data utilized in this study were obtained from the National Health Insurance Service (NHIS) database of South Korea. Due to data use agreements, access to these data is restricted, and they are not publicly available. However, researchers who satisfy the NHIS criteria for access to confidential information may request the data through the NHIS website (https://nhiss.nhis.or.kr (accessed on 30 June 2025)).

## Supplementary Materials

The following supporting information can be downloaded at: https://www.mdpi.com/article/10.3390/cancers17132262/s1, Table S1: Baseline characteristics of the study population according to glycemic status; Table S2. Risk of Bladder Cancer According to Detailed Smoking Status (Never, Former, and Current Smokers); Table S3: Dose-response association between glycemic status and bladder cancer risk in men and women according to smoking pack-years; Figure S1: Age-standardized incidence rate of bladder cancer in men and women according to glycemic and smoking status.
