# Supplementary material for: Sex-Specific Associations of Glycemic Status and Smoking with Bladder Cancer Risk: A Nationwide Cohort Study
Source: Cancers (Basel). 2025 Jul 7;17(13):2262. doi: 10.3390/cancers17132262 (PMC12249375; doi:10.3390/cancers17132262)
Supplement: Supplementary file 1 [file cancers-17-02262-s001.zip › cancers-3699629-supplementary.pdf]

Table S1. Baseline characteristics of the study population according to glycemic status.

	Men			*P* value	Women			*P* value
	Normoglycemia (n = 3,360,831)	Prediabetes (n = 1,339,469)	Diabetes (n = 504,454)		Normoglycemia (n = 3,144,799)	Prediabetes (n = 823,067)	Diabetes (n = 319,711)	
Age (years), mean ± SD	43.3±13.3	47.7±12.8	54.9±11.9	<0.001	46.7±14.2	52.8±13.3	60.9±11.3	<0.001
Anthropometrics, mean ± SD								
BMI (kg/m^2)	23.9±3.0	24.6±4.0	25.0±3.1	<0.001	22.8±3.2	24.1±4.3	25.2±3.6	<0.001
Waist circumference (cm)	82.6±8.1	84.7±8.1	87.0±8.3	<0.001	75.0±8.9	78.7±9.2	83.3±9.2	<0.001
Systolic BP (mmHg)	123.0±13.5	127.0±14.4	129.5±15.5	<0.001	117.9±15.1	124.0±16.0	128.9±16.5	<0.001
Diastolic BP (mmHg)	77.2±9.4	79.5±9.9	80.0±10.2	<0.001	73.3±9.9	76.6±10.3	77.9±10.2	<0.001
Laboratory findings, mean ± SD								
Fasting glucose (mg/dL)	87.7±7.8	108.0±6.6	150.1±51.1	<0.001	87.3±7.6	107.3±6.4	141.7±46.9	<0.001
Total cholesterol (mg/dL)	192.5±38.7	200.1±43.4	195.2±47.8	<0.001	193.3±40.2	206.0±44.5	202.5±48.8	<0.001
HDL-cholesterol (mg/dL)	54.1±32.3	53.8±31.2	51.5±33.0	<0.001	60.9±34.7	59.1±33.6	55.1±37.4	<0.001
LDL-cholesterol (mg/dL)	111.7±38.6	114.3±38.9	106.8±43.2	<0.001	113.5±37.7	123.0±38.6	117.2±42.6	<0.001
eGFR (mL/min/1.73 m^2)	89.3±54.7	85.6±37.9	85.0±38.9	<0.001	88.4±36.7	84.4±28.9	80.9±29.9	<0.001
Alcohol consumption[a], n (%)				<0.001				<0.001
None	994,717 (29.6)	366,550 (27.4)	179,464 (35.6)		2,261,912 (71.9)	624,764 (75.9)	280,193 (87.6)	
Light-to-moderate	1,937,190 (57.6)	750,041 (56.0)	242,295 (48.0)		846,429 (26.9)	187,925 (22.8)	37,402 (11.7)	
Heavy	428,924 (12.8)	222,878 (16.6)	82,695 (16.4)		36,458 (1.2)	10,378 (1.3)	2,116 (0.7)	
Smoking status, n (%)				<0.001				<0.001
Never	1,042,497 (31.0)	404,475 (30.2)	157,359 (31.2)		2,985,479 (94.9)	784,826 (95.4)	305,400 (95.5)	
Ever	2,318,334 (69.0)	934,994 (69.8)	347,095 (68.8)		159,320 (5.1)	38,241 (4.6)	14,311 (4.5)	
Regular exercise, n (%)	649,550 (19.3)	275,714 (20.6)	122,027 (24.2)	<0.001	478,043 (15.2)	134,800 (16.4)	56,945 (17.8)	<0.001
Low-income status, n (%)	495,643 (14.8)	187,253 (14.0)	87,615 (17.4)	<0.001	660,664 (21.0)	174,922 (21.3)	63,658 (19.9)	<0.001
Comorbidities, n (%)								
Hypertension	675,397 (20.1)	433,714 (32.4)	277,677 (55.1)	<0.001	588,807 (18.7)	279,995 (34.0)	196,614 (61.5)	<0.001
Dyslipidemia	426,907 (12.7)	257,769 (19.2)	180,168 (35.7)	<0.001	482,492 (15.3)	224,732 (27.3)	157,990 (49.4)	<0.001
Chronic kidney disease	169,092 (5.0)	90,614 (6.8)	49,286 (9.8)	<0.001	206,741 (6.6)	77,992 (9.5)	53,954 (16.9)	<0.001
Hypoglycemic medications								
Insulin	0 (0)	0 (0)	37,453 (7.4)	-	0 (0)	0 (0)	33,725 (10.6)	-
Sulfonylurea	0 (0)	0 (0)	234,073 (46.4)	-	0 (0)	0 (0)	181,561 (56.8)	-
Metformin	0 (0)	0 (0)	225,964 (44.8)	-	0(0)	0 (0)	178,355 (55.8)	-
Thiazolidinedione	0 (0)	0 (0)	42,021 (8.3)	-	0 (0)	0 (0)	29,609 (9.3)	-
Dipeptidyl peptidase 4 inhibitors	0 (0)	0 (0)	25,331 (5.0)	-	0 (0)	0 (0)	19,711 (6.2)	-

BMI, body mass index; BP, blood pressure; eGFR, estimated glomerular filtration rate; HDL, high-density lipoprotein; LDL, low-density lipoprotein; SD, standard deviation.

Table S2. Risk of Bladder Cancer According to Detailed Smoking Status (Never, Former, and Current Smokers)

Smoking status	Glycemic status	n	Event, n	Person-years	Age-standardized IR*	Hazard ratio (95% CI)		
						Model 1	Model 2	Model 3
Never								
	Normoglycemia	4,027,976	3,231	33,378,570	9.7	1 (Reference)	1 (Reference)	1 (Reference)
	Prediabetes	1,189,301	1,603	9,796,963	16.4	1.05 (0.99-1.12)	1.06 (0.99-1.12)	1.03 (0.97-1.10)
	Diabetes	462,759	1,171	3,713,856	31.5	1.27 (1.19-1.36)	1.27 (1.19-1.36)	1.20 (1.12-1.29)
Former								
	Normoglycemia	800,431	1,742	6,595,724	26.4	1.27 (1.20-1.36)	1.29 (1.21-1.37)	1.28 (1.20-1.36)
	Prediabetes	369,290	1,097	3,026,617	36.2	1.39 (1.30-1.50)	1.41 (1.31-1.52)	1.37 (1.28-1.48)
	Diabetes	145,841	803	1,162,733	69.1	1.74 (1.61-1.89)	1.75 (1.62-1.90)	1.65 (1.52-1.79)
Current								
	Normoglycemia	1,677,223	2,693	13,805,681	19.5	1.67 (1.58-1.77)	1.70 (1.60-1.79)	1.71 (1.61-1.81)
	Prediabetes	603,945	1,323	4,930,888	26.8	1.76 (1.64-1.88)	1.79 (1.67-1.91)	1.77 (1.65-1.89)
	Diabetes	215,565	899	1,710,675	52.6	2.12 (1.96-2.29)	2.15 (1.99-2.32)	2.05 (1.90-2.21)

* Age-standardized IR of bladder cancer per 100,000 person-years. Model 1: adjusted for age and sex. Model 2: adjusted for age, sex, alcohol consumption, physical activity, and income. Model 3: adjusted for age, sex, alcohol consumption, physical activity, income, hypertension, dyslipidemia, chronic kidney disease, urinary tract infection, and body mass index. CI, confidence interval; IR, incidence rate.

Table S3. Dose-response association between glycemic status and bladder cancer risk in men and women according to smoking pack-years.

Smoking pack-years	Glycemic status	n	Event, n	Person-years	Age-standardized IR*	Hazard ratio (95% CI)		
						Model 1	Model 2	Model 3
**Men**								
Never								
	Normoglycemia	1,042,497	1,892	8,588,613	33.7	1 (Reference)	1 (Reference)	1 (Reference)
	Prediabetes	404,475	1,024	3,306,484	34.5	1.03 (0.95–1.11)	1.03 (0.95–1.11)	1.01 (0.94–1.09)
	Diabetes	157,359	764	1,240,844	42.7	1.28 (1.17–1.39)	1.28 (1.17–1.39)	1.22 (1.12–1.32)
<20								
	Normoglycemia	1,639,148	1,671	13,571,072	40.1	1.21 (1.13–1.29)	1.22 (1.14–1.30)	1.22 (1.14–1.31)
	Prediabetes	581,611	918	4,784,721	44.3	1.33 (1.23–1.44)	1.35 (1.24–1.46)	1.33 (1.22–1.44)
	Diabetes	159,646	538	1,284,110	53.4	1.61 (1.46–1.77)	1.62 (1.47–1.78)	1.55 (1.40–1.70)
≥20								
	Normoglycemia	679,186	2,693	5,517,281	57.1	1.71 (1.62–1.82)	1.73 (1.63–1.83)	1.73 (1.63–1.84)
	Prediabetes	353,383	1,465	2,860,359	57.7	1.73 (1.62–1.86)	1.75 (1.63–1.88)	1.72 (1.61–1.85)
	Diabetes	187,449	1,130	1,475,900	68.9	2.08 (1.93–2.24)	2.09 (1.94–2.25)	1.99 (1.85–2.15)
**Women**								
Never								
	Normoglycemia	2,985,479	1,339	24,789,957	8.2	1 (Reference)	1 (Reference)	1 (Reference)
	Prediabetes	784,826	579	6,490,479	9.4	1.15 (1.04–1.27)	1.15 (1.04–1.27)	1.12 (1.02–1.24)
	Diabetes	305,400	407	2,473,012	11.1	1.36 (1.22–1.53)	1.36 (1.21–1.52)	1.27 (1.14–1.43)
<20								
	Normoglycemia	149,182	51	1,231,630	10.3	1.24 (0.94–1.64)	1.28 (0.97–1.70)	1.28 (0.97–1.70)
	Prediabetes	34,027	30	279,043	16.5	1.99 (1.39–2.86)	2.05 (1.43–2.96)	2.01 (1.39–2.89)
	Diabetes	11,330	17	90,526	16.2	1.96 (1.21–3.16)	2.00 (1.24–3.23)	1.88 (1.16–3.03)
≥20								
	Normoglycemia	10,138	20	81,422	18.4	2.22 (1.43–3.45)	2.26 (1.45–3.53)	2.26 (1.45–3.53)
	Prediabetes	4,214	7	33,382	14.3	1.73 (0.82–3.64)	1.77 (0.84–3.73)	1.72 (0.82–3.63)
	Diabetes	2,981	17	22,872	44.5	5.42 (3.36–8.75)	5.48 (3.40–8.86)	5.16 (3.19–8.34)

* Age-standardized IR of bladder cancer per 100,000 person-years. Model 1: adjusted for age. Model 2: adjusted for age, alcohol consumption, physical activity, and income. Model 3: adjusted for age, alcohol consumption, physical activity, income, hypertension, dyslipidemia, chronic kidney disease, urinary tract infection, and body mass index. CI, confidence interval; IR, incidence rate.

.

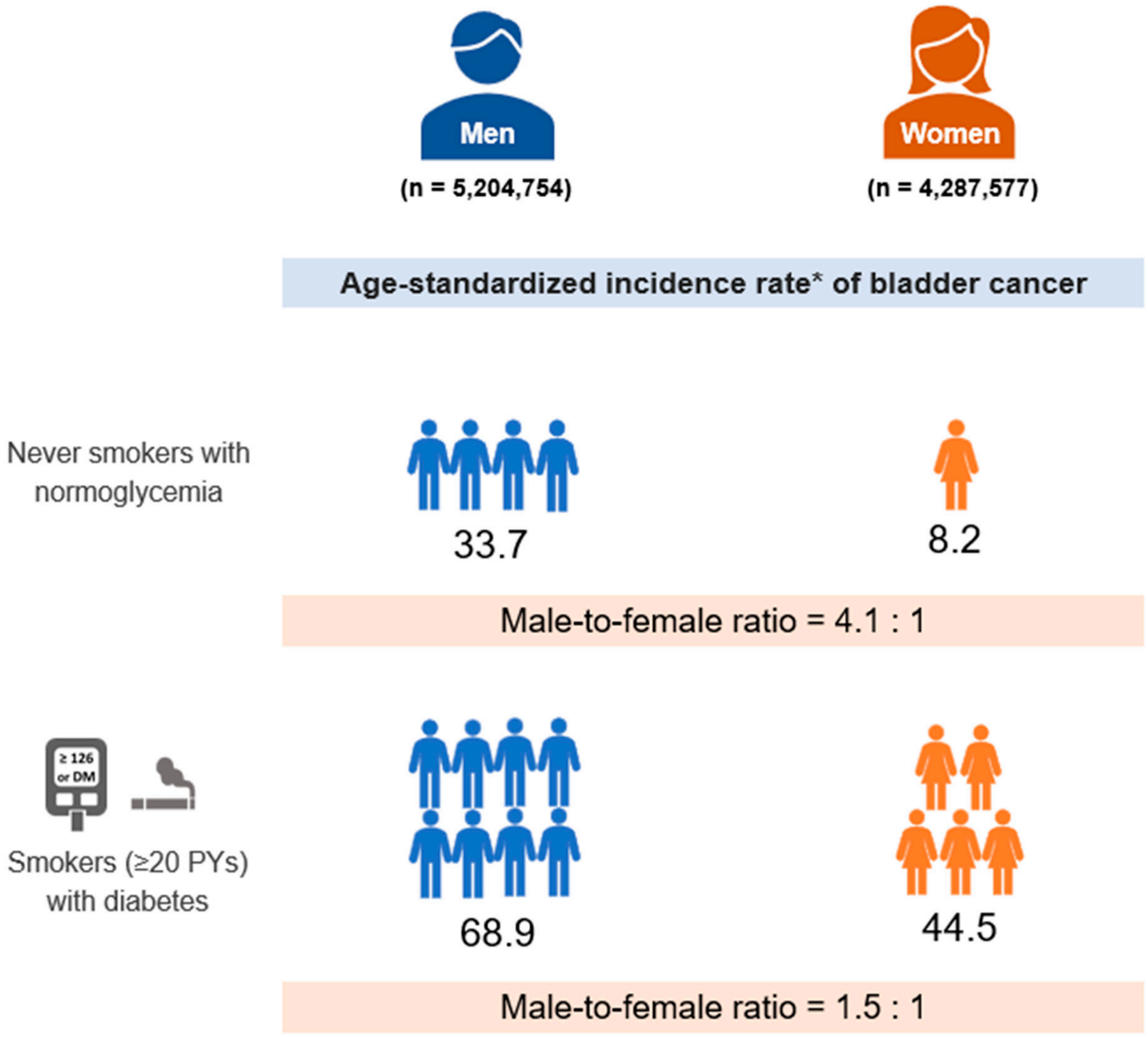

Figure S1. Age-standardized incidence rate of bladder cancer in men and women according to glycemic and smoking status. The male-to-female ratio of bladder cancer incidence decreased as glycemic status worsened and pack-years of smoking increased. *Age-standardized incidence rate per 100,000 person-years. PY, pack-years.
